# Publisher Correction: Temporal control of gene expression by the pioneer factor Zelda through transient interactions in hubs

**DOI:** 10.1038/s41467-019-08346-3

**Published:** 2019-01-15

**Authors:** Jeremy Dufourt, Antonio Trullo, Jennifer Hunter, Carola Fernandez, Jorge Lazaro, Matthieu Dejean, Lucas Morales, Saida Nait-Amer, Katharine N. Schulz, Melissa M. Harrison, Cyril Favard, Ovidiu Radulescu, Mounia Lagha

**Affiliations:** 1 Institut de Génétique Moléculaire de Montpellier, University of Montpellier, CNRS-UMR 5535, 1919 Route de Mende, 34293 cedex 5 Montpellier, France; 2 Department of Biomolecular Chemistry, University of Wisconsin School of Medicine and Public Health Madison, 6204B Biochemical Sciences Building 440 Henry Mall, 53706 Madison, WI USA; 3 Institut de Recherche en Infectiologie de Montpellier, CNRS, University of Montpellier UMR 9004, 1919 route de Mende, 34293 cedex 5 Montpellier, France; 4 DIMNP, UMR CNRS 5235, University of Montpellier, Place E. Bataillon – Bât. 24 cc 107, 34095 cedex 5 Montpellier, France

Correction to: *Nature Communications* 10.1038/s41467-018-07613-z; published online 05 December 2018.

The original version of this Article contained an error in Fig. 4a, in which the “=” sign of the equation was inadvertently replaced with a “-“ sign. This has been corrected in the PDF and HTML versions of the Article.

