# Supplementary material for: Temporal control of gene expression by the pioneer factor Zelda through transient interactions in hubs
Source: Nat Commun. 2018 Dec 5;9:5194. doi: 10.1038/s41467-018-07613-z (PMC6281682; doi:10.1038/s41467-018-07613-z)
Supplement: Supplementary file 1 — Supplementary Information [file 41467_2018_7613_MOESM1_ESM.pdf]

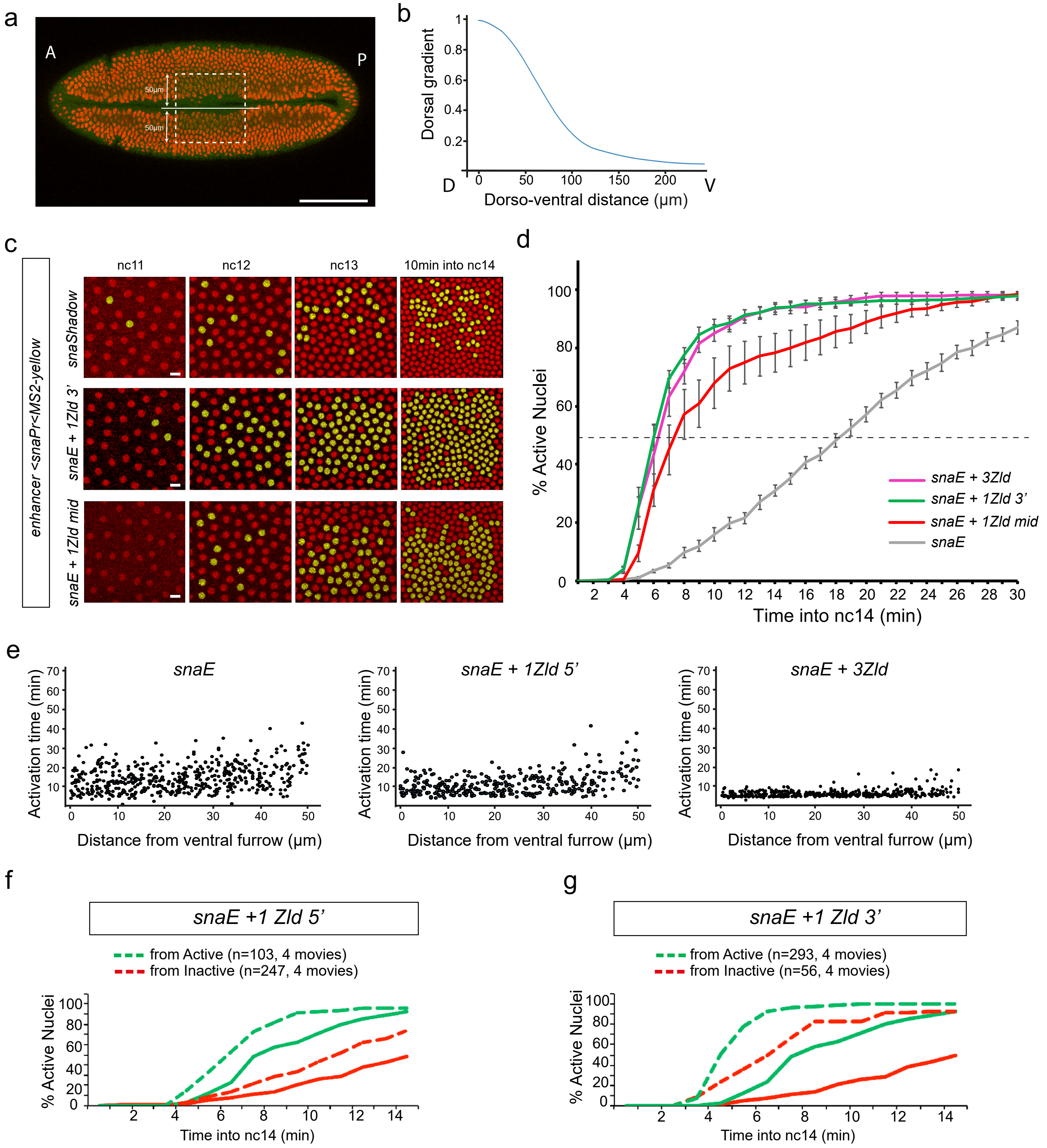

Supplementary Figure 1: Zelda-binding sites provide quantitative temporal control of gene expression (related to Figure 1)
(**a**) Maximum intensity projected Z-stacks of a representative gastrulating embryo tilescan showing the imaged region (dashed line) related to Figure 1. Nuclei are in red and MS2 dots in green. The embryo is oriented with Anterior (A) to the left, and Posterior (P) to the right. Scale bars represent 100µm. (**b**) Schematic representation of the Dorsal gradient along the Dorso-ventral axis (D-V), based on[1]. (**c**) Snapshots of maximum intensity projected Z-stacks from live-imaging movies of transcriptional activity driven by *sna-shadow*, *snaE+1Zld 3'* and *snaE+1Zld mid* transgenes from nc11 to nc14. Nuclei exhibiting transcriptional activity were false-colored in yellow. Scale bars represent 10µm. (**d**) Synchrony curve for *snaE+1Zld 3'* (green line), *snaE+1Zld mid* (red line), *snaE+3Zld* (purple line) transgenes compared to that of the *snaE* (grey line) transgene. Statistics: *snaE* (6 movies, n=970 nuclei), *sna-shadow* (4 movies, n=612 nuclei), *snaE+3Zld* (4 movies, n=824 nuclei), *snaE+1zld* mid (4 movies, n=659 nuclei). Error bars represent SEM. (**e**) Scatter plot of the activation time as a function of the distance from the ventral furrow at nc14, for *snaE* (left panel), *for snaE+1Zld5'* (middle panel) and for *snaE+3Zld* (right panel) transgenic embryos. Statistics: *snaE* (6 movies, n=490 nuclei), *snaE+1Zld5'* (4 movies, n=350 nuclei), *snaE+3Zld* (4 movies, n=411 nuclei). (**f-g**) Quantification of transcriptional memory in *snaE* (**f-g**), *snaE+1Zld 5'* (**f**) and *snaE+1Zld 3'* (**g**) transgenic embryos. Temporal dynamics from *snaE* are shown as solid curves, while those from extra Zld binding sites transgenes are represented with dashed curves (**f-g**). Each graph shows the percentage of transcriptionally active nuclei in the first 15min of nc14 for nuclei coming from active nc13 mothers (green curves) and inactive nc13 mothers (red curves). Only nuclei located 50µm around the ventral furrow are considered.

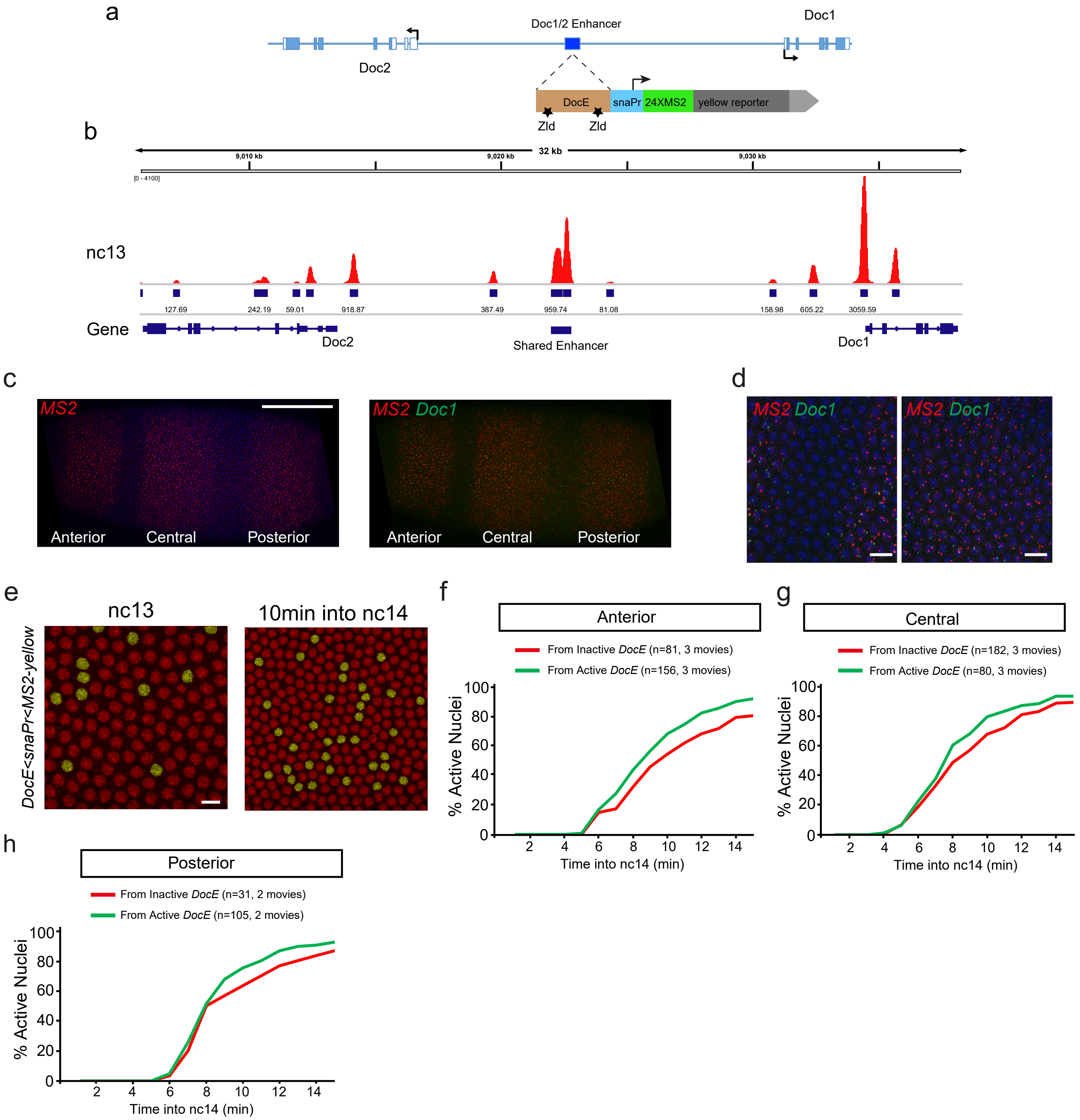

Supplementary Figure 2: Transcriptional dynamics from a *Doc* enhancer
(**a**) Schematic representation of the *Doc1/2* locus and localization of the regulatory element (*Doc-Enhancer*) used in this Figure. (**b**) The *Doc-Enhancer* was designed according to Zelda ChIP data on nc13 embryos[2]. A transgenic line was created with the *Doc-Enhancer* driving expression of an *MS2-yellow* reporter from a *sna* minimal promoter (*DocE transgene*) (**a**).
(**c**) A nc14 *DocE* transgenic embryo was hybridized with a probe against *MS2* (red) and an intronic probe against endogenous *Doc1* (green). Maximum intensity projected Z-stacks confocal images with MS2 signal alone (left panel) or merged with *Doc1* (right panel). The *DocE* transgene drives expression in the dorsal mesoderm in a tripartite pattern (anterior, central and posterior), scale bar represent 100µm. (**d**) Zoomed images from the embryo shown in (**c**), scale bars represent 10µm. (**e**) Snapshots from a maximum intensity projected Z-stack movie of a *DocE* transgenic embryo, imaged in the central dorsal ectoderm domain. Nuclei exhibiting an active transcriptional dot are false colored in yellow. The *DocE* transgene leads to a stochastic expression in nc13, which allows transcriptionally active mothers to be distinguished from their inactive neighbors. Scale bar represent 10µm. (**f-h**) Quantification of transcriptional memory in *DocE* transgenic embryos in three regions of the dorsal ectoderm, anterior (3 movies, n=237 nuclei) (**f**), central (3 movies, n=262 nuclei) (**g**) and posterior (2 movies, n=136 nuclei) (**h**).

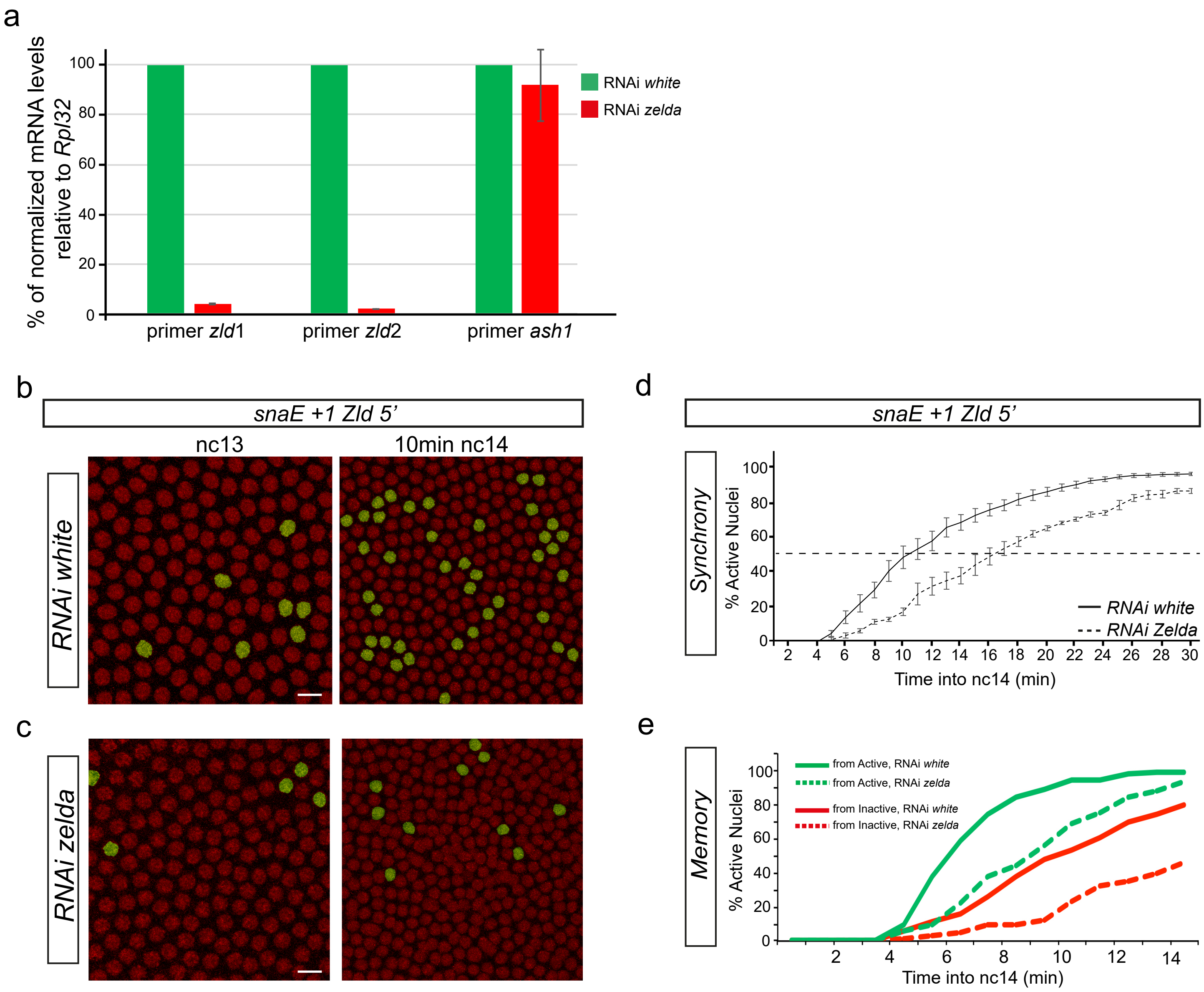

Supplementary Figure 3: Transcriptional memory persists in the absence of Zelda (related to Figure 3)
(**a**) mRNA quantification using qRT-PCR in *white-RNAi* and *zld-RNAi* 0-2-hr embryos. *RpL32* was used as a control mRNA for normalization, *ash1* was used as a control mRNA. The levels of mRNA for *zld* and *ash1* in *zld-RNAi* embryonic extracts (red) were normalized to their levels in *white-RNAi* extracts to 100% (green). For each genotype, experiments were performed on four biological replicates. Two primer pairs were used to quantify *zld* mRNA (zld1 and zld2). Error bars represent SEM. (**b, c**) Snapshots of maximum intensity projected Z-stacks *Drosophila* embryo movies expressing the *snaE+1Zld5'* transgene at nc13 or at nc14 in a *white-RNAi* (**b**) or in a *zld-RNAi* genetic background (**c**). Nuclei with active transcription spots are false colored in yellow.
(**d**) Temporal coordination for the *snaE+1Zld5'* transgene in a *white-RNAi* (solid curves) and in a *zld-RNAi* genetic background (dashed curves). Statistics: *SnaE+1Zld5' white-RNAi* (5 movies, n=452 nuclei) and *snaE+1Zld5' zld-RNAi* (5 movies n=167 nuclei). (**e**) Kinetics of activation during the first 15min of nc14 driven by the *snaE+1Zld5'* transgene in *white-RNAi* (solid curves) or *zld-RNAi* embryos (dashed curves). The kinetic of nuclei coming from transcriptionally active mother nuclei in nc13 (green curves) is compared to those arising from inactive mothers (red curves). In Supplementary Fig. 3, only nuclei located within a 25µm rectangle from 'pseudo ventral furrow' are considered. Statistics: *SnaE+1Zld5' white-RNAi* (5 movies, n=228 nuclei) and *snaE+1Zld5' zld-RNAi* (5 movies n=97 nuclei). Scale bars represent 10µm.

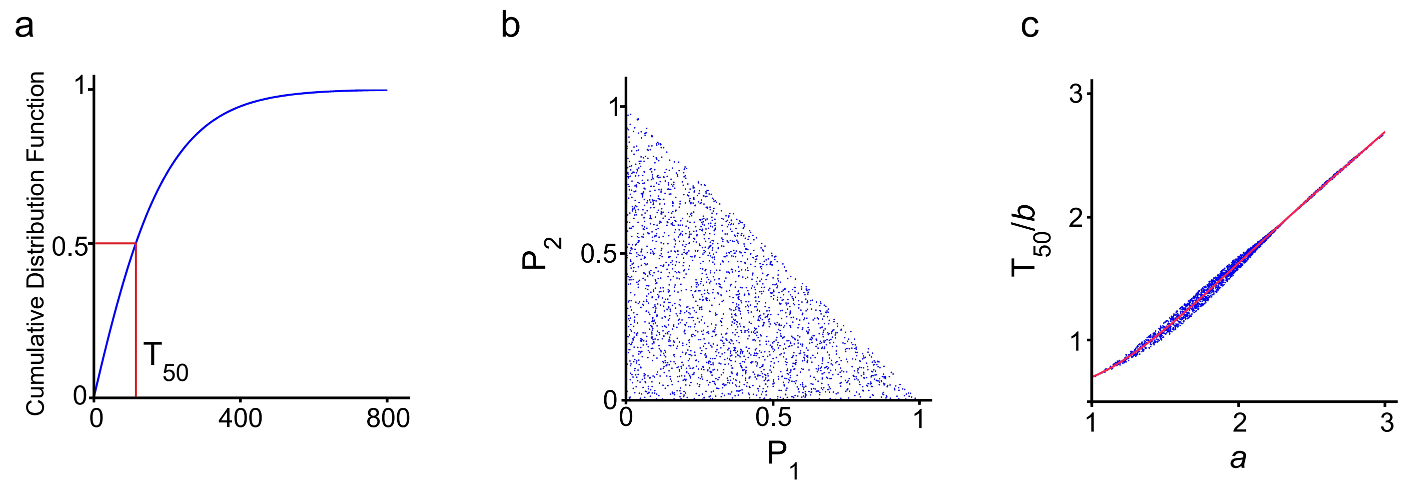

Supplementary Figure 4: Mathematical modeling of transcriptional memory (related to Figure 4)
(**a-c**) Graphs resulting from simulated data from the homogeneous jump model. (**a**) We defined T_{50} as the time needed to reach 50% of total activation; the inverse of this time is thus a measure of the speed of reactivation. Using the homogeneous jump model, we computed the random component of this time for many values of *'b'* and for many values of p_1 and p_2, (probabilities to reach activation in one and two jumps, respectively).
(**b**) Panel showing the uniformly distributed values of p_1 and p_2. (**c**) When projected onto the plane of the variables *'a'* and T_{50}/b (blue dots), the simulated data appear close to the curve of equation:T_{50}/b= 0.085a4−0.7834a3+2.651a2−2.814a+1.56 (red line).
These findings imply that the activation time (T_{50}) is proportional to *'b'* and increases with *'a'*. Furthermore, in units of *'b'*, this time depends on *'a'* only.

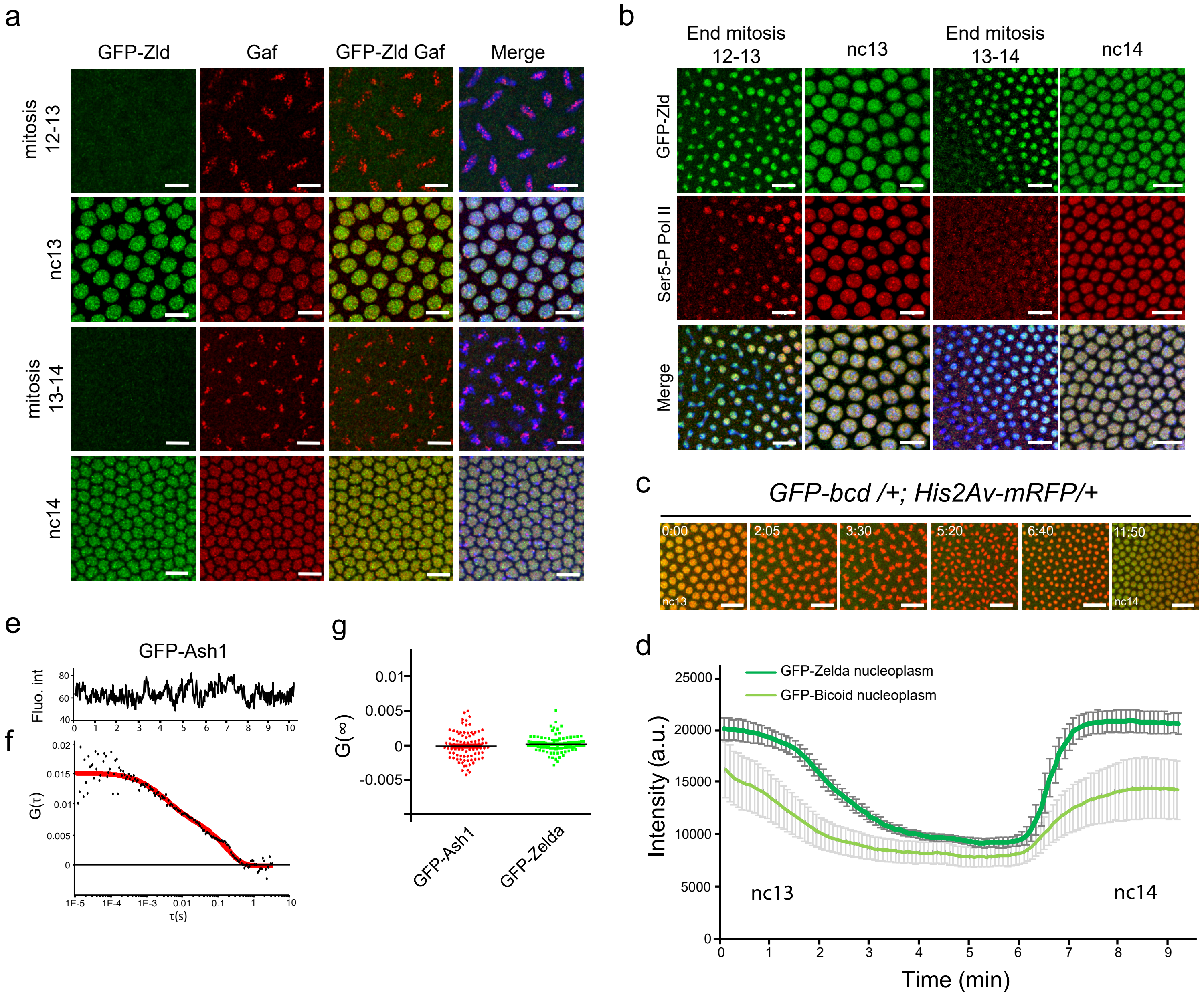

Supplementary Figure 5: Zelda global kinetic properties (related to Figure 5)

(**a**) Contrary to GAF and as previously shown[3], Zelda is not obviously retained on mitotic chromosomes. Immunostaining of *GFP-zld* embryos confocal images with anti-GFP (green) and anti-GAF (red) are shown at different time points: mitosis between nc12 and nc13 (top row panels), interphase nc13 (second row panels), mitosis between nc13 and nc14 (third row panels) and interphase nc14 (bottom row panels). Scale bars represent 10µm.

(**b**) Immunostaining using anti-GFP and anti-Ser5-P Pol II in *GFP-zld* embryo from the end of mitosis between nc12 and nc13 to nc14 showing that Zelda comes back very quickly in the nucleus at the end of mitosis compared to Ser5-P Pol II. Scale bars represent 10µm.

(**c**) Living *eGFP-bcd/+;His2Av-mRFP/+* embryo imaged by confocal microscopy from interphase of nc13 to early interphase of nc14. Successive representative maximum intensity projected Z-stack images are shown at the indicated timings. Scale bars represent 20µm.

(**d**) Average intensity profiles for nucleoplasmic GFP-Zelda (dark green) and nucleoplasmic eGFP-Bicoid (light green) measured from a nc13 embryo transitioning into nc14. An automatic tracking of fluorescence from a minimum of 87 nuclei generated these profiles, error bars represent SD. Synchronization of the developmental timing was done using the time frame where mother nuclei are splitting into two daughter nuclei, using His2Av-mRFP staining.

(**e**) Example of a time trace obtained by FCS from a *GFP-ash1* nc14 embryo that showed no bleaching. (**f**) Example of autocorrelation function (black dots) related to (**e**) (red curves represent fitting using diffusion reaction model). (**g**) Dot plot representing values for G(∞) (see Equation 10). Centered lines represent the median.

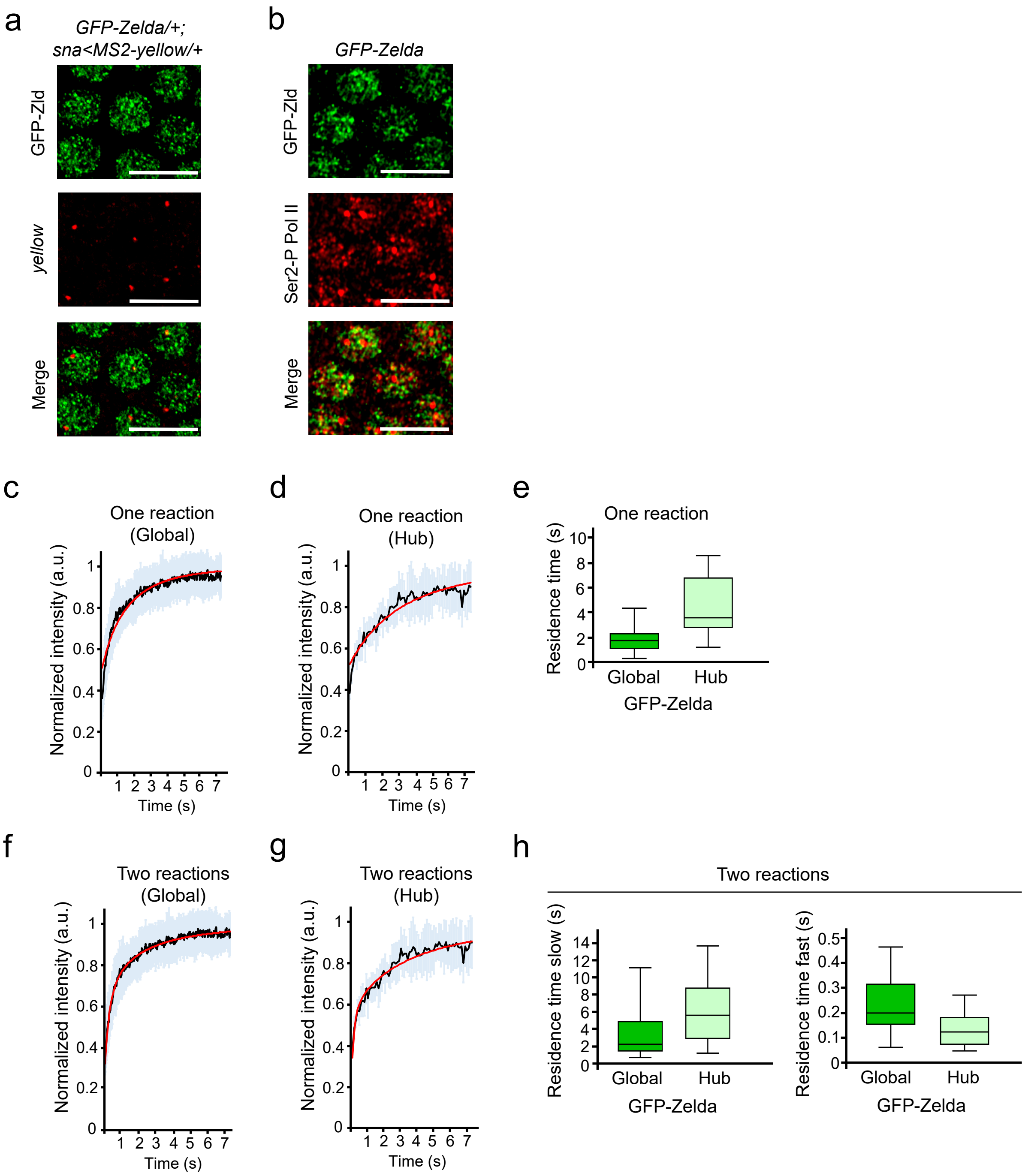

i

Fitting models		Residence time Global (s)	Residence time Hub (s)
Reaction only		1.9 +/- 1	4.3 +/- 2.2
Diffusion reactions		3.3 +/- 2.8	2.5 +/- 1.6
Two reactions	slow	3.2 +/- 2.3	5.7 +/- 3.7
	fast	0.23 +/- 0.1	0.14 +/- 0.07

Supplementary Figure 6: Zelda hubs kinetic properties (related to Figure 6)
(a) Immuno-FISH using anti-GFP antibody (green) and probes against *yellow* (red) in *GFP-Zld* embryos expressing the *SnaE+1Zld3'* transgene showing limited colocalization between Zelda hubs and transcription site. Scale bars represent 10µm. (**b**) Immunostaining using anti-GFP (green) and anti-Ser2-P Pol II (red) antibodies in *GFP-Zld* embryos showing no obvious colocalization between Zelda hubs and Pol II. Scale bars represent 10µm. (**c**) Fluorescence Recovery After Photobleaching (FRAP) mean curve (black) and the mean of all the fits (red curve) using one reaction models determined at the bleached spot (Global) for 25 nuclei from nc14 developing *GFP-zld* embryos. The error bars represent SD from different nuclei. (**d**) FRAP mean curve (black) and the mean of all the fit (red curve) using one reaction models determined at the bleached spot (Hub) for 11 nuclei from nc10-13 developing *GFP-zld* embryos. The error bars represent SD from different nuclei. (**e**) Box plot representing the extracted residence time between Global FRAP and Hub FRAP using one reaction model. Centered lines represent the median and whiskers represent min and max. (**f**) FRAP mean curve (black) and the mean of all the fits (red curve) using two reaction model determined at the bleached spot (Global) for 25 nuclei from nc14 developing *GFP-zld* embryos. The error bars represent SD from different nuclei. (**g**) FRAP mean curve (black) and the mean of all the fit (red curve) using two reaction model determined at the bleached spot (Hub) for 11 nuclei from nc10-13 developing *GFP-zld* embryos. The error bars represent SD from different nuclei. (**h**) Box plot representing the two extracted residence time (slow and fast) between Global FRAP and Hub FRAP using two reaction model. Centered lines represent the median and whiskers represent min and max. (**i**) Table showing the mean residence time +/- SD using the three fitting models for Global or Hub FRAP.

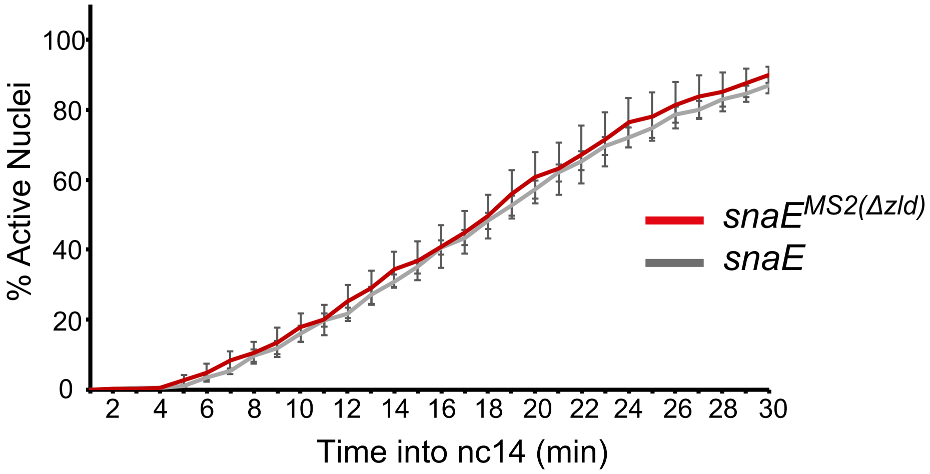

Supplementary Figure 7: Synchrony profile from *snaE* revealed by an alternative MS2 sequence.
Synchrony curve for *snaE* with *MS2deltaZld* (red line)[4] transgene compared to that of the *snaE* (grey line) transgene. Statistics: *snaE* (6 movies, n=970 nuclei), *snaE* with *MS2deltaZld* (3 movies, n=456 nuclei). Error bars represent SEM.

Supplementary methods:

Homogeneous jump model

In this model, all jumps have the same mean duration, denoted τ. The distribution of the random time T_r depends on τ and on the number of transitions performed in order to reach the ON state. If only one transition is utilized to reach the ON state, then T_r is exponentially distributed with the parameter τ^{-1}. Likewise, in the case where the ON state is reached after performing k transitions, then T_r is gamma distributed with shape parameter k and with scale parameter τ. In general, T_r is given by a mixture of gamma distributions with shape parameters 1, 2, 3, ... and scale parameter τ, whose cumulative distribution function (cdf) reads:

$$F_r(t) = P[T_r \leqslant t] = p_1\left(1 - \exp\left(-t/\tau\right)\right) + p_2 \frac{1}{\Gamma(2)} \gamma\left(2, t/\tau\right) + p_3 \frac{1}{\Gamma(3)} \gamma\left(3, t/\tau\right) + \cdots, \tag{1}$$

where p_1, p_2, p_3 are the probabilities of one, two, and three jumps, Γ, γ are the complete and incomplete gamma functions, respectively. The mean and the variance of T_r are as such:

$$E[T_r] = (p_1 + 2p_2 + 3p_3 + \ldots)\tau \tag{2}$$
$$Var[T_r] = (p_1 + 2p_2 + 3p_3 + \ldots)\tau^2$$

We define the following parameters of the mixed distribution that can be computed empirically from the mean and the variance, the first two moments of the distribution, are as such:

$$b = Var\,[T_r]/E\,[T_r], \qquad a = (E[T_r])^2/Var\,[T_r] \tag{3}$$

Using Supplementary Equation 2 we find that:

$$b = \tau, \qquad a = p_1 + 2p_2 + 3p_3 + \cdots \tag{4}$$

showing that the parameter *'a'* represents the average number of transitions and, equivalently, the mean shape parameter of the mixed gamma distribution, whereas *'b'* is the mean transition time.

In the case of three jumps, the model has three independent parameters b, p_1, p_2. The parameter *'a'* can be computed with Supplementary Equation 4 and p_3 follows from $p_1+p_2+p_3=1$. The synchrony of re-activation can be quantified by the time needed for activating half of the population. We denote this time T_{50} and we can compute it as the solution of the equation $F_r(T_{50})=0.5$. Supplementary Equation 1

shows that F_r depends on the rescaled argument t/b, also on p_1, p_2. Therefore T_{50}/b=ψ where ψ does not depend on *'b'* but depends on p_1, p_2. By sampling uniformly the possible values of p_1, p_2 we showed (see Supplementary Fig. 4) that ψ depends on p_1, p_2 essentially via *'a'*, and that the following approximate relation holds:

$$T_{50} = b\psi(a), \tag{5}$$

where, for the three jumps model, $\psi(a) = 0.085a^4 - 0.78a^3 + 2.65a^2 - 2.81a + 1.56$, and $1 \leq$ *'a'* ≤ 3. Supplementary Equation 5 implies that population activation can be delayed either by increasing *'b'* (the transition time) or by increasing *'a'* (the number of transitions).

Heterogeneous jump model

In this model, the mean jump durations are heterogeneous. Thus, for a three states model, in order to reach the ON state, one can perform a single jump ($A_3 \rightarrow$ON in mean time b_3), two jumps ($A_2 \rightarrow A_3$, then $A_3 \rightarrow$ON, in mean times b_2 and b_3), or three jumps ($A_1 \rightarrow A_2$, then $A_2 \rightarrow A_3$, then $A_3 \rightarrow$ON, in mean times b_1, b_2 and b_3). For uneven mean jump durations, $b_1 \neq b_2 \neq b_3$, the time to perform two or three jumps is no longer gamma distributed. However, even in this case, it is possible to compute the cdf of T_r. To this aim, we consider the continuous time Markov chain $M(t)$, that has four states A_1, A_2, A_3, A_4 = ON, and three transitions $A_1 \overset{k_1}{\rightarrow} A_2, A_2 \overset{k_2}{\rightarrow} A_3,\ A_3 \overset{k_3}{\rightarrow} ON,$ where the transitions rates are reciprocals of the mean transition times, namely $k_i = b_i^{-1}$, i=1,2,3. The ON state is absorbing, i.e. once it reaches this state, the process remains there. Then, the activation time T_r results as a solution to the first passage time problem. More precisely, let $T_j = min(t > 0|M(t) = ON, M(0) = A_j)$ be the first passage time in ON when $M(t)$ starts from A_j, where j=1, 2, or 3. If p_1, p_2, p_3 are the probabilities of starting in A_1, A_2, or A_3, respectively, then one has

$$P[T_r \leq t] = p_1 P[T_1 \leq t] + p_2 P[T_2 \leq t] + p_3 P[T_3 \leq t] \tag{6}$$

Let $X_{ij}(t) = P[M(t) = A_i|M(0) = A_j]$. Because the state ON is absorbing,

$$X_{4j}(t) = P\left[M(t) = ON|M(0) = A_j\right] = P[T_j \leq t] \tag{7}$$

Furthermore, because $M(t)$ is a Markov chain, each of the vectors $X_{ij}(t) = (X_{1j}(t), X_{2j}(t), X_{3j}(t), X_{4j}(t))$ satisfies the following system of ordinary differential equations (master equation):

$$\frac{dX}{dt} = QX, \qquad (8)$$

where

$$Q = \begin{pmatrix} -\frac{1}{b_1} & 0 & 0 & 0 \\ \frac{1}{b_1} & -\frac{1}{b_2} & 0 & 0 \\ 0 & \frac{1}{b_2} & -\frac{1}{b_3} & 0 \\ 0 & 0 & \frac{1}{b_3} & 0 \end{pmatrix}$$

The solution of Supplementary Equation 8 is $X(t) = exp(Qt)X(0)$, where $exp(Qt) = 1 + Qt + (Q^2t^2)/2! + \ldots$ The corresponding initial conditions are $X_1(0) = (1, 0, 0, 0)$, $X_2(0) = (0, 1, 0, 0)$, $X_3(0) = (0, 0, 1, 0)$ and $X_4(0) = (0, 0, 0, 1)$. Next, it can be easily shown that

$$\exp(Qt) =$$

$$\begin{pmatrix} e^{-\frac{t}{b_1}} & 0 & 0 & 0 \\ \frac{b_2}{b_1-b_2}\left(e^{-\frac{t}{b_1}} - e^{-\frac{t}{b_2}}\right) & e^{-\frac{t}{b_2}} & 0 & 0 \\ \frac{b_1b_2b_3\left(e^{-\frac{t}{b_1}}-e^{-\frac{t}{b_2}}\right)-b_1b_3^2\left(e^{-\frac{t}{b_1}}-e^{-\frac{t}{b_3}}\right)+b_2b_3^2\left(e^{-\frac{t}{b_2}}-e^{-\frac{t}{b_3}}\right)}{(b_1-b_2)(b_1-b_3)(b_2-b_3)} & \frac{b_3\left(e^{-\frac{t}{b_1}}-e^{-\frac{t}{b_2}}\right)}{b_2-b_3} & e^{-\frac{t}{b_3}} & 0 \\ 1-\frac{b_1^2b_2e^{-\frac{t}{b_1}}-b_1b_2^2e^{-\frac{t}{b_2}}+b_1b_3^2e^{-\frac{t}{b_3}}+b_2^3b_3e^{-\frac{t}{b_2}}-b_2b_3^2e^{-\frac{t}{b_3}}}{(b_1-b_2)(b_1-b_3)(b_2-b_3)} & 1-\frac{b_2e^{-\frac{t}{b_2}}-b_3e^{-\frac{t}{b_3}}}{b_2-b_3} & 1-e^{-\frac{t}{b_3}} & 1 \end{pmatrix}. \qquad (9)$$

Using Supplementary Equation 7 and Supplementary Equation 9 it follows

$$P[T_3 \le t] = X_{43}(t) = (0,0,0,1)\, exp(Qt)\, (0,0,1,0)^T = 1 - e^{-\frac{t}{b_3}},$$

$$P[T_2 \le t] = X_{42}(t) = (0,0,0,1)\, exp(Qt)\, (0,1,0,0)^T = 1 - \frac{b_2e^{-\frac{t}{b_2}} - b_3e^{-\frac{t}{b_3}}}{b_2 - b_3},$$

$$P[T_1 \le t] = X_{41}(t) = (0,0,0,1)\, exp(Qt)\, (1,0,0,0)^T$$

$$= 1 - \frac{b_1^2b_2e^{-\frac{t}{b_1}} - b_1b_2^2e^{-\frac{t}{b_2}} + b_1b_3^2e^{-\frac{t}{b_3}} + b_2^3b_3e^{-\frac{t}{b_2}} - b_2b_3^2e^{-\frac{t}{b_3}}}{(b_1 - b_2)(b_1 - b_3)(b_2 - b_3)},$$

where $(\ )^T$ stands for transpose.

Finally, using Supplementary Equation 6 we obtain

$$F_r(t) = p_1\left(1 - \frac{b_1^2 b_2 e^{-\frac{t}{b_1}} - b_1 b_2^2 e^{-\frac{t}{b_2}} + b_1 b_3^2 e^{-\frac{t}{b_3}} + b_2^3 b_3 e^{-\frac{t}{b_2}} - b_2 b_3^2 e^{-\frac{t}{b_3}}}{(b_1-b_2)(b_1-b_3)(b_2-b_3)}\right) + p_2\left(1 - \frac{b_2 e^{-\frac{t}{b_2}} - b_3 e^{-\frac{t}{b_3}}}{b_2-b_3}\right) + p_3\left(1 - e^{-\frac{t}{b_3}}\right) \quad (10)$$

Modelling memory and memory erasure

Our model explains the part of memory resulting from differences of *'a'* (number of jumps needed to reach the ON state) between descendants of active and inactive mothers. We simulated activation in nc14 by considering that, before mitosis, active mothers are in A_3, whereas inactive mothers are in A_1. During mitosis (5 minutes), the ON state is not accessible and downward transitions $A_3 \rightarrow A_2$ and $A_2 \rightarrow A_1$ are possible. After mitosis, the state is transmitted to the daughters that further follow the previously described irreversible transition scheme. We have simulated this model and computed the ratio $a_{inactive}/a_{\mathrm{active}}$ corresponding to the number of steps needed to reach the ON state after mitosis for nuclei coming from inactive and active mothers, respectively. We noticed that the post-mitotic ratio is smaller than the pre-mitotic value (which is 3), the reduction resulting from mitotic transitions between non-productive states. Furthermore, we computed the dependence of the post-mitotic ratio on the transition time *'b'* and showed that this ratio increases with *'b'*, it is equal to one for small *'b'*, and it is larger than one for large *'b'*. This result is robust with respect to the simulation parameters. The free parameter in the simulation is the ratio of transition times for upward and backward transitions during mitosis. The theoretical curve in the Fig. 4g was obtained when backward transitions are two times slower than upward transitions.

Data analysis and parameter estimates for modeling

Data analysis and parameter estimates were performed using MATLAB and Optimization Toolbox Release 2013b, The MathWorks, Inc., Natick, Massachusetts, United States.

The time origin was first set at the end of mitosis. The deterministic waiting time $T0$ was estimated as the time between the end of mitosis and the time when the first nucleus from a large population is activated. This estimate is accurate for a

large number of nuclei, because the probability p_0 that T_r is close to zero was supposed to be non-zero. The estimate is more accurate when the number of nuclei is large (by the law of large numbers) and when the number of transitions is small (because p_0 is high for a gamma distribution with small shape parameter, as it is the case when the number of transitions is small). Then the origin of time was set at T_0 and T_r was determined for all nuclei. Parameters *'a'* and *'b'* were first, roughly estimated with the formulas in Supplementary Equation 3. We found that *'a'* is not higher than 3, which allowed us to restrict the analysis to only three unidirectional transitions.

The empirical cumulative distribution function of T_r was estimated using the Kaplan-Meier method. The homogeneous jump model in Supplementary Equation 1 and the heterogeneous jump model in Supplementary Equation 10 were fitted by minimization of an objective function O defined as the l^2 (sum of squares) distance between experimental data and model prediction. The optimization was performed using the MATLAB function *lsqnonlin* starting from 100, different, randomly chosen initial parameter values. The best optimum and the suboptimal solutions where the objective function was at most 50% higher than the best optimum were used to quantify parameter uncertainty. For instance, if the optimal and suboptimal values of the parameter p are between p_{min} and p_{max}, its uncertainty is computed as

$$err_p = \frac{p_{max}-p_{min}}{p_{max}+p_{min}} \qquad (11)$$

The results of the fit are the three parameters p_1, p_2, p_3 and *'b'* (Supplementary Data 1). The parameter *'a'* is also computed using Supplementary Equation 4. The values of *'a'* and *'b'* estimated from Supplementary Equation 3 and from the distribution fit can be different. The first method, based on the moments, is sensitive to extreme values; therefore, it is less reliable than the latter.

The results of the parameter estimation are given in the Supplementary Data 1 and 2. For Fig. 4c-g and Supplementary Data 1 and 2, after checking that single genotype estimates were equivalent to the pooled data, we decided to present the estimates for *'a'* population of nuclei pooled from *Zelda-RNAi snaE* and *Zelda-RNAi snaE+1Zld 5'* embryos. Given that the only difference between *snaE* and *snaE+1Zld 5'* transgenes resides in the added CAGGTAG, we reasoned that in the absence of Zelda, these genotypes are theoretically comparable, thus justifying their pooling, to increase sample size.

Supplementary References

1. Rushlow, C. A. & Shvartsman, S. Y. Temporal dynamics, spatial range, and transcriptional interpretation of the Dorsal morphogen gradient. *Current Opinion in Genetics & Development* **22,** 542–546 (2012).
2. Harrison, M. M., Li, X.-Y., Kaplan, T., Botchan, M. R. & Eisen, M. B. Zelda Binding in the Early Drosophila melanogaster Embryo Marks Regions Subsequently Activated at the Maternal-to-Zygotic Transition. *PLoS Genet* **7,** e1002266 (2011).
3. Raff, J. W., Kellum, R. & Alberts, B. The Drosophila GAGA transcription factor is associated with specific regions of heterochromatin throughout the cell cycle. *The EMBO Journal* **13,** 5977–5983 (1994).
4. Lucas, T. *et al.* 3 minutes to precisely measure morphogen concentration. (2018). doi:10.1101/305516
