# Supplementary material for: Temporal control of gene expression by the pioneer factor Zelda through transient interactions in hubs
Source: Nat Commun. 2018 Dec 5;9:5194. doi: 10.1038/s41467-018-07613-z (PMC6281682; doi:10.1038/s41467-018-07613-z)
Supplement: Supplementary file 3 — Description of Additional Supplementary Files [file 41467_2018_7613_MOESM3_ESM.pdf]

Supplementary Movie 1: Live imaging of transcriptional activity driven by *snaE*. Nuclei are detected using His2Av-mRFP and MS2 using MCP-GFP.

Supplementary Movie 2: Live imaging of transcriptional activity driven by *snaE+1Zld 5'*. Nuclei are detected using His2Av-mRFP and MS2 using MCP-GFP.

Supplementary Movie 3: Live imaging of transcriptional activity driven by *snaE+2Zld*. Nuclei are detected using His2Av-mRFP and MS2 using MCP-GFP.

Supplementary Movie 4: Live imaging of transcriptional activity driven by *snaE+3Zld*. Nuclei are detected using His2Av-mRFP and MS2 using MCP-GFP.

Supplementary Movie 5: Live imaging of transcriptional activity driven by *snaE+3Zld* in a *white-RNAi* background. Nuclei are detected using His2Av-mRFP and MS2 using MCP-GFP.

Supplementary Movie 6: Live imaging of transcriptional activity driven by *snaE+3Zld* in a *zelda-RNAi* background. Nuclei are detected using His2Av-mRFP and MS2 using MCP-GFP.

Supplementary Movie 7: Live imaging of transcriptional activity driven by *docE* in a *white-RNAi* background. Nuclei are detected using His2Av-mRFP and MS2 using MCP-GFP.

Supplementary Movie 8: Live imaging of transcriptional activity driven by *docE* in a *zelda-RNAi* background. Nuclei are detected using His2Av-mRFP and MS2 using MCP-GFP.

Supplementary Movie 9: Living *GFP-zld/+;His2Av-mRFP/+* embryo imaged by confocal microscopy.

Supplementary Movie 10: Living e*GFP-bcd/+;His2Av-mRFP/+* embryo imaged by confocal microscopy.

Supplementary Movie 11: Live imaging of *GFP-zelda* embryo from nc11 to nc14 showing local inhomogeneities.

Supplementary Movie 12: Example of GFP-Zld hubs and their fast movements.

Supplementary Movie 13: Live imaging of Zelda (green) in a *GFP-zelda* embryo containing the *snaE+1Zld3'* transgene and the MCP-RFPt (MS2 in Red).

Supplementary Movie 14: Example of Fluorescence Recovery After Photobleaching (FRAP) of one GFP-Zld hub, showing fast movement and fast recovery.

File Name: Supplementary Data 1
Description: Results of the fit of the homogeneous jump model to our live imaging data. This model has 3 free parameters ‘b’, ‘p1’ and ‘p2’. The parameter ‘a’ was computed with Supplementary Equation 3. The parameter 'p3' is obtained from p1+p2+p3=1. All the parameters were fit into two conditions: from inactive mothers (red) and from active mothers (green). The relative uncertainty due to multiple local optima is provided in percentage, for each parameter. The goodness of fit is given by the sum of squares distance O.

File Name: Supplementary Data 2
Description: Results of the fit of the heterogeneous jump model to our live imaging data. This model has 5 free parameters ‘p1’, ’p2’, ‘b1’, ‘b2’, and ‘b3’. The parameter 'p3' is obtained from p1+p2+p3=1. The parameter ‘a’ was computed with Supplementary Equation 3. All the parameters were fit into two conditions: from inactive mothers (red) and from active mothers (green). The relative uncertainty due to multiple local optima is provided in percentage, for each parameter. The goodness of fit is given by the sum of squares distance O.

File Name: Supplementary Data 3
Description: Primers.

1919 route de Mende, 34293 Montpellier cedex 5
Tel : (33) 04 34 35 96 50 – Fax : (33) 04 34 35 96 34 – Web : http://www.igmm.cnrs.fr/
e-mail : mounia.lagha@igmm.cnrs.fr
